# Mental health technology tools: two alternative approaches

**DOI:** 10.1017/S2045796020000025

**Published:** 2020-02-03

**Authors:** Robert E. Drake

**Affiliations:** Westat, Lebanon, New Hampshire, USA

Health researchers propose that information technology, artificial intelligence and robotics will dominate health care at some undetermined point, but probably sooner than we expect. Mental health will be no exception: Information technology is the future. Researchers are currently developing electronic assessments, scientific algorithms for treatment, virtual therapists and electronic self-help tools. One current dilemma concerns how to make use of the thousands of electronic tools that are already available to help users manage their health conditions. As the two editorials in this section state, clear guidelines for users, clinicians and researchers do not exist. How do clinicians know which tools to recommend? How should mental health programmes incorporate technology tools? What should researchers be developing and studying? The two following editorials offer different pathways.

John Torous and Aditya Vaidyam argue that a single mental health app may be what is needed and that the underlying electronic structure is the key. They and their colleagues at Harvard have been designing and testing such a tool, called mindLAMP, which has many advantages in terms of flexibility, data security, usability and other features. Elizabeth Carpenter-Song describes a different pathway, which she and her colleagues at Dartmouth have been studying. She argues that the current state of confusion requires a technology specialist to help users identify their individual goals, find the most appropriate tools to facilitate achieving those goals and learn to use the tools. Because so many tools are available, many of which address appropriate goals other than symptoms, a technology specialist may be needed, at least in the near term. Both arguments are compelling and should be of interest to mental health service users, clinicians and researchers.

As investigators develop and study these two approaches, they may want to consider several aspects, including but not limited to the following. A first consideration is to determine how clinicians should be involved. Some people with mental health concerns will of course prefer to maintain privacy by using tools on their own, but many others will be engaged in the treatment of one kind or another such that coordination becomes salient. Because we know that fragmented care results in different, sometimes conflicting messages and produces poor outcomes, integration is preferable. But how should this be implemented? Clinicians are often uninformed and sometimes resistant to new technologies. Perhaps either approach (using monothetic or polythetic tools) should include a process of educating and linking the user and provider together to ensure that both understand the technology and work collaboratively to optimise effectiveness.

Second, the legal, ethical and financial issues related to technology tools must be addressed. Who bears legal responsibility for monitoring the use and effects of these tools? What is the clinician's obligation to partner in an informed fashion? And how will the time allotted to education, technology support and collaboration be paid for? These are all unanswered questions.

Finally, research on rapidly developing technologies is challenging. By the time that investigators go through the traditional research procedures, such as pilot testing, writing a grant and doing a series of studies, the technology and content will have evolved. It remains true, however, that nothing substitutes adequately for empirical validation. Eminence-based, expert-based and profit-based models of medical care have failed repeatedly, by producing no effects or significant harms. Technology products are often marketed without any empirical evidence, and procedures for rapid, unbiased testing are needed. Researchers, in concert with funders, should develop new ways of proceeding to protect the public from harm and enhance effectiveness. The pace of development is likely to quicken, and the need is already urgent.

